# Corticospinal function does not predict stride-to-stride gait dynamics in older adults

**DOI:** 10.3389/fnetp.2026.1946459

**Published:** 2026-10-09

**Authors:** J. S. Gomes, J. H. Oliveira, P. Bauer, L. Sousa, A. Sel, N. Cortes, J. R. Vaz

**Affiliations:** 1 Egas Moniz Center for Interdisciplinary Research (CiiEM), Egas Moniz School of Health & Science, Almada, Portugal; 2 Neuromuscular Research Laboratory, Faculty of Human Kinetics, University of Lisbon, Lisbon, Portugal; 3 CIPER, Faculty of Human Kinetics, University of Lisbon, Lisbon, Portugal; 4 Centre for Brain Science, Department of Psychology, University of Essex, Colchester, United Kingdom; 5 School of Sport, Rehabilitation and Exercise Sciences, University of Essex, Colchester, United Kingdom

**Keywords:** ageing, corticospinal drive, gait adaptability, stride-to-stride fluctuations, transcranial magnetic stimulation, network physiology

## Abstract

**Introduction:**

Ageing is associated with impaired gait adaptability, which leads to an increased fall risk in older adults. The study of the temporal structure of stride‐to‐stride fluctuations may help mitigate these inherent risks, as it has been proposed as a marker of locomotor functionality and motor control. However, the underlying mechanisms responsible for the progressive deterioration of stride‐to‐stride fluctuations in older adults are yet to be fully unravelled. Recent research suggests several central and peripheral factors can influence the walking adaptation process with the corticospinal tract playing a key role in motor control during gait. Thus, the purpose of this study was to investigate whether lower‐leg corticospinal drive significantly explains the temporal structure of gait stride-to-stride fluctuations.

**Methods:**

Thirty‐four older adults underwent transcranial magnetic stimulation (TMS) assessments of the tibialis anterior in one experimental session, where corticospinal excitability (CSE), silent period duration (SP), and short‐interval intracortical inhibition (SICI) were quantified. Immediately after the TMS intervention, they also performed a 12‐min treadmill walking trial at preferred walking speed, during which inter‐stride intervals were extracted and analysed using detrended fluctuation analysis to obtain the fractal scaling exponent (αISIs). Multiple linear regression analysis was used to examine whether CSE, SP and SICI predicted αISIs.

**Results:**

The results of the linear regression model indicated that none of the corticospinal measures significantly predicted gait’s temporal dynamics (F(3,28) = 0.20, *p* = 0.896, *R* ^2^ = 0.021).

**Discussion:**

These findings suggest that gait adaptability does not seem to be explained by corticospinal function, likely emerging from distributed, multilevel neural control processes involving spinal, supraspinal, and peripheral mechanisms rather than corticospinal contributions alone.

## Introduction

Older adults frequently experience movement detriments as they age, which often lead to falls, serious injuries, hospitalizations or even death (Hausdorff et al., 2001; Colón-Emeric et al., 2024). These movement detriments, such as shortened stride lengths or impaired balance during locomotion, have been shown to result from these individuals’ reduced ability to flexibly adapt their locomotion patterns in response to challenges or constraints in their environment (Hausdorff et al., 1997). Our ability to navigate and move around is heavily dependent on the complex interactions involving multiple systems such as the central nervous system, the musculoskeletal system and the endocrine systems, which allow behavioural adaptation in response to the environmental demands (Bartsch et al., 2015; Cavanaugh et al., 2017). Notably, these systems, which are crucial for behavioural adaptation, undergo age-related declines that are associated with movement deficits and increased movement variability (Hausdorff et al., 1997). Among these changes, alterations in the corticospinal tract may represent a key neurophysiological mechanism underlying the reduced capacity to adapt movement patterns to changing environmental demands, as supraspinal centres have been suggested to play an important role in the control of human gait (Barthélemy et al., 2011; Nielsen, 2003). Therefore, it is essential to understand the underlying mechanisms responsible for these movement deficits to provide effective preventive and rehabilitative care for those at risk.

In the past two decades, research has pinpointed movement variability as an inherent component of the biological system central to the movement adaptation process (Buzzi et al., 2003; Stergiou et al., 2006; Stergiou and Decker, 2011). Such variability is defined as the variation in the performance of an action across multiple repetitions of the same task (Stergiou et al., 2006). While some authors considered movement variability to solely reflect random error (i.e., noise), more recent frameworks suggest that variability over time provides key information concerning the motor control process underlying human movement (Stergiou, 2018; Stergiou et al., 2006). A good example of this biological quality is the occurrence of natural stride-to-stride fluctuations during a gait task, emerging as means to achieve a stable walking pattern (Hausdorff, 2005). It appears then that the structure of variability over time is critical to the human being’s capability to react and adapt to changes in the environment (Cavanaugh et al., 2017; Harbourne and Stergiou, 2009; Ravi et al., 2021; Stergiou et al., 2006). While the structure of these natural stride-to-stride fluctuations is thought to be flexibly adaptive in healthy individuals, indicating a rich and compliant behavioural state (Stergiou et al., 2006), a breakdown of this structure is evident in older adults (Hausdorff et al., 2003; Lipsitz and Goldberger, 1992; Manor et al., 2010). Importantly, this breakdown is associated with a deterioration of an individual’s adaptive abilities (Lipsitz and Goldberger, 1992; Stergiou et al., 2006).

Successful gait depends on the nervous system’s ability to produce coordinated, rhythmic alternation between flexion and extension of the limbs and to adapt this fundamental walking pattern in response to sensory input, such as visual cues, especially when encountering unfamiliar or unpredictable environments (Spedden et al., 2019). Accordingly, various central and peripheral factors can influence the walking adaptation process (Spedden et al., 2019). While some studies have primarily examined peripheral contributors to gait detriments among older adults, such as sarcopenia (Landi et al., 2012), sensory nerve loss (Lipsitz et al., 2018), or joint stiffness (Watson et al., 2011), supraspinal centres have been suggested to play an important role in the control of human gait (Nielsen, 2003). The corticospinal tract is of particular interest as it has been suggested to be heavily involved in the control of walking (Barthélemy et al., 2011; Nielsen, 2003; Petersen et al., 2001). Petersen and colleagues (2001) demonstrated that electromyographical (EMG) activity of the *tibialis anterior* and *soleus* muscles during walking was supressed by changes in motor evoked potentials, specifically inhibitory cortical interneurons, highlighting the relevant role of the intracortical inhibitory motor networks in muscle control during walking. Barthelemy and Nielsen (2010) also demonstrated a suppression of EMG activity during walking, but for the *posterior deltoid* muscle, responsible for the swing of the arms, likely resulting from the motor evoked inhibition of intracortical interneurons. Nonetheless, a deeper understanding of the neural mechanisms underlying gait adaptability in older adults is critical, particularly to understand its connection with the inherent movement deficits in these individuals.

Ageing is associated with functional alterations in the corticospinal pathway, particularly affecting the neural control of ankle muscles (Spedden et al., 2018). Corticospinal contributions during gait have been studied through several techniques, such as transcranial magnetic stimulation (TMS) and coherence analysis of the coupling between electroencephalographic (EEG) and EMG signals (Barthélemy et al., 2012; Nielsen, 2003; Petersen et al., 2012). Ageing appears to be accompanied by a reduction in corticospinal drive during locomotion, which may compromise the ability to perform adaptive and anticipatory adjustments in gait (Roeder et al., 2018; Sato and Choi, 2022; Spedden et al., 2019). It has been suggested that this decline in neural drive may contribute to reduced sensorimotor adaptability, a factor increasingly recognized as a key determinant of mobility limitations in older adults (Fettrow et al., 2023). However, conflicting findings preclude an adequate interpretation of corticospinal behaviour in older adults as some authors observed decreased corticospinal excitability (McGinley et al., 2010; Oliviero et al., 2006) while others suggested no age-related changes (Rozand et al., 2019). Intracortical inhibitory mechanisms of the primary motor cortex are also the subject of contrasting results, as McGinley and colleagues (2010) observed increased intracortical inhibition while others reported a decrease (Christie and Kamen, 2014; Oliviero et al., 2006). Therefore, it is critical to advance our knowledge regarding the behaviour of corticospinal drive in older adults’ gait, further understanding its role in modulating the diminished adaptability these individuals present.

Swanson and Fling (2018) have recently proposed a more complex interaction between motor adaptability and neural information: despite age-related declines in gait coordination, older adults exhibiting greater motor cortex inhibition demonstrated superior walking performance (measured through the phase coordination index, which measures the degree of consistency and accuracy in generating a series of anti-phase left-right stepping phases). These findings point toward a potential age-related shift in the functional role of motor cortex inhibition in lower limb control. Thus, ageing is associated to a shift of the neural representation of movement control from motor related cortical and subcortical regions, and the spinal cord, towards premotor cortical regions (Fettrow et al., 2023). Collectively, these observations support the notion that ageing alters the neural representations and network dynamics of gait control, likely reflecting a decline in the interplay among neural and musculoskeletal subsystems that underpin adaptable locomotion. Nonetheless, the extent to which changes in stride-to-stride temporal dynamics are linked to alterations in cortical and corticospinal inputs remains unclear. Addressing this gap is critical, as a deeper understanding of the neural mechanisms driving age-related changes in gait control and adaptability could guide future research towards improving rehabilitation protocols and interventions.

The purpose of the present study was to investigate if lower leg corticospinal drive was a significant predictor of the temporal structure of older adults’ stride-to-stride fluctuations. Accordingly, we hypothesised the temporal structure of stride-to-stride fluctuations would be explained by lower leg corticospinal drive, asserting the inherent age-related neural decrements are determinant for gait’s motor control.

## Materials and methods

### Participants

Thirty-four older adults (17 males, 17 females; age: 69.76 ± 7.06 years; height: 1.66 ± 0.09 m; body mass: 73.36 ± 14.81 kg; body mass index: 26.59 ± 4.22 kg/m^-2^) took part in the present study. Participants were excluded if they had any neurological or neuromuscular disorders, implanted medical devices (e.g., cardiac pacemakers, cochlear implants, or medical pumps), were taking medications that could affect nervous system function or presented any other contraindications to the use of transcranial magnetic stimulation (TMS) technique. Prior to participating in the experimental procedures, all individuals provided written informed consent and completed a TMS safety screening questionnaire (Keel et al., 2001). Participants were asked to visit the laboratory on one occasion for an experimental session with a duration of ∼1 h. Participants were instructed to arrive at the laboratory well hydrated, abstain from alcohol for 24 h, and avoid strenuous physical activity for 48 h before the experimental session. This study was conducted at Egas Moniz School of Health and Science, approved by the Ethics Committees of Egas Moniz School of Health and Science (#1378), and conducted in accordance with the Declaration of Helsinki.

### Experimental procedures

The experimental session initiated with participants performing two maximal voluntary isometric contractions (MVICs) of dorsiflexion, each lasting 5 s and separated by a 60-s rest period. They were instructed to generate maximal force as quickly as possible while receiving strong verbal encouragement. MVIC force was defined as the highest torque value recorded during each trial. If the difference between trials exceeded 5%, additional trials were performed until two consecutive trials differed by ≤ 5%. The highest MVIC obtained was then used to establish submaximal force targets. Immediately after, participants underwent assessment of cortical and corticospinal responses through transcranial magnetic stimulation (TMS). Afterwards, participants underwent preferred walking speed (PWS) determination. Briefly, PWS was calculated by gradually increasing the speed of the treadmill in increments of 0.2 m/s. Participants were asked to indicate when the speed felt comfortable. Once a comfortable speed was reached, additional increments of 0.2 m/s were applied until the participant reported that the speed was “too fast to be comfortable.” The same procedure was then used to decrease the speed until the participant indicated that it was “too slow to be comfortable.” This process was repeated three times in each direction, and the average of the six measurements was used to determine PWS (Vaz et al., 2024). Subsequently, participants performed a 12min gait task at the respective PWS, from which the temporal structure of stride-to-stride fluctuations (αISIs) was collected. Figure 1 illustrates the experimental session.

**FIGURE 1:**
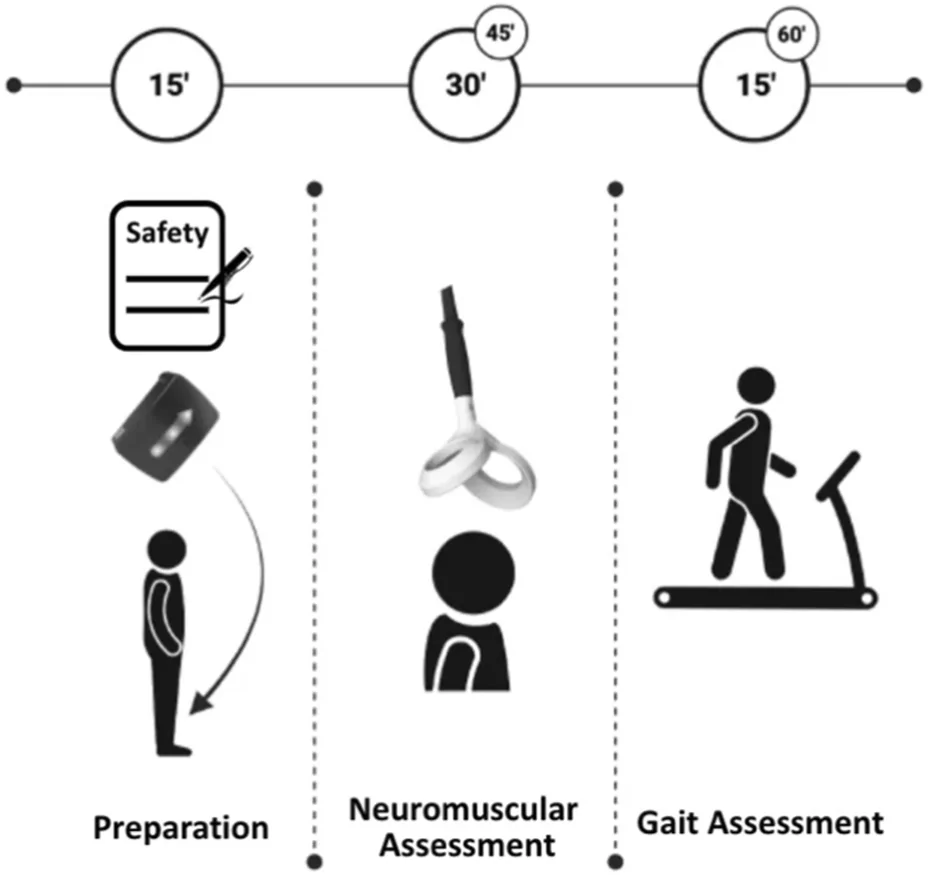
Schematic representation of the experimental session.

### Isokinetic dynamometry and electromyography

All force and neuromuscular assessments were performed using an isokinetic dynamometer (Humac Norm, CSMi, Stoughton, MA, USA), which was initialised and calibrated according to the manufacturer’s instructions. Participants were seated in the isokinetic dynamometer with the hip and knee of their dominant leg set at 90° and 120°, respectively (Tallent et al., 2012; Thompson et al., 2011). The foot of the dominant leg was firmly strapped into the ankle adapter of the dynamometer whilst the knee was secured in a thigh stabiliser, and additional straps were firmly fastened across the participant’s shoulders and waist to prevent any extraneous movement of the upper leg. The dynamometer seat was adjusted to ensure proper alignment between the participant’s lateral malleolus and the dynamometer axis of rotation. Prior to testing, participants performed several submaximal isometric knee extension contractions to ensure adequate muscle warm-up. All measurements were obtained with their ankle plantar flexed at 110° (20° past the anatomical zero; Hirano et al., 2015; 2018; Thompson et al., 2011).

Surface electromyographic (EMG) activity of the dorsiflexor and plantarflexor muscles was recorded using bipolar surface electrodes (Trigno Avanti, Delsys, Natick, MA, USA). Prior to electrode placement, the skin was prepared by shaving, lightly abrading, and cleaning the area with an alcohol swab. Electrodes were positioned over the muscle bellies of the *tibialis anterior* (TA) and *gastrocnemius medialis* (GM) in accordance with the recommendations of the Surface EMG for Non-Invasive Assessment of Muscles (SENIAM), aligned parallel to the muscle fibres (Hermens et al., 2000). To reduce movement-related artifacts, electrodes were additionally secured with adhesive tape. EMG signals were amplified (x 1000) and band-pass filtered between 20 and 450 Hz using a Trigno Avanti EMG amplifier (Delsys, Natick, MA, USA). Force and EMG data were sampled at 1000 Hz and 1266 Hz, respectively, via a CED Micro1401 data acquisition system (Cambridge Electronic Design, Cambridge, UK) interfaced with a personal computer. All data was recorded using Spike2 software (Version 7; Cambridge Electronic Design, Cambridge, UK).

### Transcranial magnetic stimulation

Single-pulse and paired pulse TMS techniques were applied over the primary motor cortex using a double-cone coil (110 mm diameter; maximum output 1.4 T) connected to a Magstim 200 magnetic stimulator (Magstim Co. Ltd., Whitland, UK). To determine the optimal site for activation of the TA, commonly referred to as “hotspot”, the coil was placed 0.5–1 cm posterior and along the anteroposterior plane of the vertex, as it has previously been suggested to be in this location (Devanne et al., 1997; Tallent et al., 2012). The coil was positioned to induce a posterior to anterior current in the underlying motor cortex, targeting the contralateral leg representation. To confirm optimal location site for the TA, the coil was moved until the largest MEP response was observed at 50% stimulator output during a contraction at 20% MVIC, as suggested by Rossini and colleagues (2015) in their updated IFCN report. Once optimal coil placement was established, the location was marked with indelible ink to ensure consistency across testing throughout the entire experimental session.

The active motor threshold (AMT), defined as the lowest stimulation intensity capable of eliciting a MEP amplitude greater than 200 µV in at least three of five consecutive trials (Kidgell and Pearce, 2010), was determined at the beginning of the session. To identify AMT, stimulation intensity was initially set at 50% of maximal stimulator output and decreased in 5% increments until the MEP amplitude no longer exceeded 200 µV. The intensity was then increased in 1% increments until the minimal intensity capable of producing MEPs greater than 200 µV in three out of five trials was identified.

Single-pulse TMS was used to assess corticospinal excitability quantified by MEP amplitude and GABA_B_-mediated intracortical inhibition through SP duration (Paci et al., 2021), whereas paired-pulse TMS was used to assess GABA_A_-mediated intracortical inhibition through SICI (Mrachacz-Kersting et al., 2021). The single-pulse protocol consisted of ten stimuli delivered at 130% AMT. The paired-pulse protocol consisted of ten paired stimuli, including a subthreshold conditioning stimulus at 80% AMT followed by a suprathreshold test stimulus at 130% AMT, with an interstimulus interval of 3 m. All stimuli were delivered during a sustained contraction at 20% MVIC, with interstimulus intervals randomly varying between 6 and 12 s to minimize anticipation of stimulation (Mason et al., 2020). The TA muscle was chosen because it is the primarily active muscle during the swing phase of gait and, with ageing, it becomes weaker and stiffer, leading to a reduced joint range of motion and a reduction of the minimum toe clearance (Perera et al., 2021). Figure 2a illustrates the neuromuscular assessment as well as the participant’s positioning on the isokinetic dynamometer.

**FIGURE 2:**
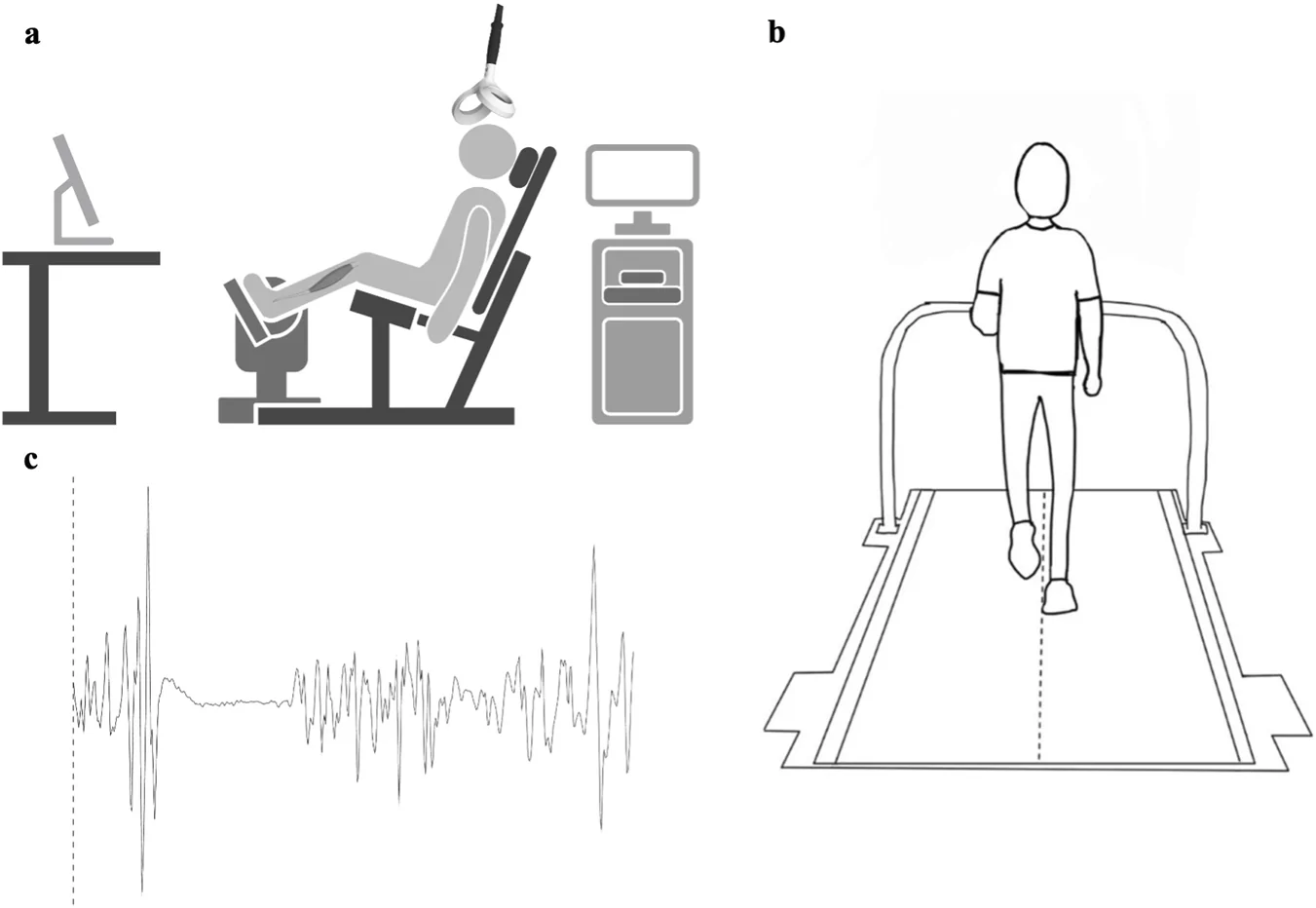
**(a)** Schematic representation of the neuromuscular assessment and the participants’ positioning on the dynamometer. **(b)** Schematic representation of the gait assessment. **(c)** Real example of a motor evoked potential (MEP) elicited during the neuromuscular assessment.

### Instrumented treadmill

The walking trials took place on an instrumented treadmill (Bertec Corporation, Columbus, OH, USA) and ground reaction forces of individuals steps were used to precisely determine heel-strike events. Synchronisation between the treadmill’s force plates and analogue devices was managed via a Vicon Lock Lab unit integrated with Vicon Nexus software (Vicon Motion Systems Ltd., Oxford, UK). Force data were collected at 1000Hz. Figure 2b provides a schematic representation of the gait assessment.

### Data analysis

All data were processed using custom Matlab® R2025b (The MathWorks, Natick, MA, USA) scripts. MVIC force was defined as the peak force value obtained during the MVIC trials of the experimental session. For statistical purposes, the highest MVIC force was used.

Peak-to-peak amplitude of MEPs were determined as the difference, in millivolts (mV), between the maximum positive and maximum negative deflection of the EMG waveform in the 100 ms after the delivery of each stimulus (Figure 2c). MEP peak-to-peak amplitude provided a measure of corticospinal excitability (CSE). GABA_B_-mediated intracortical inhibition was measured through SP duration, in milliseconds (ms), for unconditioned MEPs, calculated between the stimulus artefact and resumption of voluntary EMG (Damron et al., 2008). To quantify SICI, a measure of GABA_A_-mediated intracortical inhibition, the ratio between the conditioned MEP and the unconditioned MEP amplitudes were calculated and then multiplied by 100 (% Unconditioned MEP). The average of the ten CSE ratios, ten SP durations and ten SICI ratios measured for each participant were included in the statistical analysis. TMS data were quality-controlled at the individual-trial level. Each of the 10 repetitions acquired at each assessment was visually inspected, and repetitions were excluded when the MEP could not be clearly distinguished, when the participant mistimed the required voluntary contraction, or when the contraction did not reach the target intensity of 20% of the participant’s MVIC. The remaining valid repetitions were retained for subsequent TMS analyses. A robust determination of SP duration was not possible for two participants due to lack of data quality to reliably determine this metric. Thus, further statistical analysis was performed with data from 32 participants instead of the full sample.

Regarding walking trials’ analysis, the first 30 s of each recording were excluded to minimize transient effects associated with adjusting to treadmill speed. Heel-strike events were identified from the ground-reaction force signal using an 8–12 N force threshold, allowing precise identification of the onset of heel contact of the dominant leg, which was used consistently for all participants. When a heel-strike event was not adequately detected, the event was treated as missing and no interpolation was performed. The analysis therefore proceeded to the following reliably detected heel strike, with inter-stride intervals calculated only between validly identified events. To quantify the temporal structure of stride-to-stride fluctuations, inter-stride intervals (ISIs) were first extracted by measuring the time between consecutive heel strikes of the same foot. From the resulting ISI time series, the fractal-scaling exponent (α) was computed, representing the temporal structure of gait fluctuations. Detrended fluctuation analysis (DFA) was employed to calculate the fractal-scaling exponent, α, from the ISI time series (αISIs; Damouras et al., 2010; Hunt et al., 2014; Peng et al., 1995). DFA is a modified random-walk method designed for long-range correlated time series. The procedure begins by integrating the time series and dividing it into windows of length n. Within each window, a least-squares line is fitted, and the integrated data are detrended by subtracting this line. The root mean square (RMS) is then calculated for each window, and these RMS values are summed across the entire time series, producing F(n). This process is repeated for progressively smaller window sizes. Plotting log F(n) against log n yields a slope corresponding to the α-value. An α-value greater than 0.5 indicates positively persistent long-range correlations (i.e., increases tend to follow increases and decreases follow decreases), while an α-value below 0.5 reflects anti-persistent correlations (i.e., increases tend to follow decreases, and *vice versa*). In this study, the DFA window sizes ranged from 16 to N/9, where N represents the total number of stride intervals (Damouras et al., 2010). The reliability of the DFA estimates was supported by the use of approximately 600 consecutive inter-stride intervals per participant, consistent with recommendations for obtaining robust DFA estimates, together with high *r* ^2^ values of the log–log regressions, indicating a strong linear fit across the scaling range.

### Statistical analysis

All statistical analyses were performed in RStudio (version 4.3.1; R Core Team, 2023). To examine the association between corticospinal and intracortical measures and the temporal structure of stride-to-stride fluctuations (αISIs), a multiple linear regression model was fitted with αISIs as the dependent variable and CSE, SP and SICI as independent variables. All variables were collected at a single time point per participant; therefore, observations were treated as independent, and no random effects were included. Model assumptions were evaluated through visual inspection of diagnostic plots and adequate statistical tests. Linearity of the relationship between predictors and outcome was assessed through the plot of residuals vs. fitted values. Normality of residuals was evaluated through the observation of Q-Q plots, and the Shapiro-Wilk test, whereas homoscedasticity was analysed through the Breusch-Pagan test. Multicollinearity among predictors was assessed using Variance Inflation Factor analysis, with values greater than five considered indicative of problematic collinearity. Influential observations were assessed using leverage and Cook’s distance, with values exceeding conventional thresholds further inspected. Lastly, independence of errors was evaluated through the Durbin-Watson test. Model fit was quantified using the coefficient of determination (*R* ^2^ and adjusted *R* ^2^). Statistical significance of individual predictors was assessed using two-tailed tests with an alpha level set *a priori* to *p* < 0.05. The relationship between variables was modelled using the following linear regression equation:

$$\alpha {\text{ISIs}}_{i}=\beta_{0}+\beta_{1}{\text{CSE}}_{i}+\beta_{2}{\text{SICI}}_{i}+\beta_{3}\text{SPi}+\varepsilon_{i}$$ (1)

## Results

Table 1 provides an overview of all variables analysed.

**TABLE 1:** Gait adaptability and neuromuscular function data.

Variables	Mean ± SD	95% CI
Gait assessment		
αISIs (au)	0.79 ± 0.15	[0.74, 0.85]
*r* ^2^ (au)	0.94 ± 0.03	[0.93, 0.95]
ISIs length (n° of strides)	628 ± 63.1	[605, 651]
PWS (ms^-1^)	1.16 ± 0.17	[1.10, 1.22]
Neuromuscular assessment		
MVC (Nm^-1^)	35.7 ± 9.30	[32.3, 39.1]
AMT (% MSO)	40.4 ± 6.46	[38.0, 42.8]
CSE (mV)	0.91 ± 0.38	[0.78, 1.05]
SP (ms)	127 ± 19.5	[120, 134]
SICI (%)	91.8 ± 17.5	[85.6, 97.9]

Data are presented as mean ± standard deviation (SD) and 95% confidence intervals (95% CI). ISIs, Inter-Stride Intervals; PWS, Preferred Walking Speed; MVC, Maximum Voluntary Contraction; AMT, Active Motor Threshold; CSE, Corticospinal Excitability; SP, Silent Period; SICI, Short-Interval Intracortical Inhibition.

### Multiple linear regression

A multiple linear regression was conducted to examine whether CSE, SICI, and SP predicted αISIS. The model’s residuals followed a normal distribution (*p* = 0.207) and exhibited constant variance across all levels of independent variables (*p* = 0.104). Multicollinearity of predictors was not present (CSE = 1.039, SP = 1.015, SICI = 1.037) and further analysis also showed errors to be independent from each other (D-W = 2.19). The overall model based on Equation 1 was not statistically significant, F(3, 28) = 0.20, *p* = 0.895, explaining *R* ^2^ = 0.021 (adjusted *R* ^2^ = −0.084) of the variance in αISIS. None of the predictors were statistically significant: CSE (β = −0.035, SE = 0.074, t = −0.48, *p* = 0.637), SICI (β = −0.0008, SE = 0.002, t = −0.52, *p* = 0.608), and SP (β = 0.0004, SE = 0.001, t = 0.26, *p* = 0.794). These findings suggest that, within this sample, CSE, SICI, and SP do not significantly predict variability in αISIS.

## Discussion

This study aimed to investigate if lower leg corticospinal drive was a significant predictor of the temporal structure of older adults’ stride-to-stride fluctuations. The present study’s findings did not support our hypothesis. We observed that the temporal structure of stride-to-stride fluctuations was not significantly predicted by lower leg corticospinal drive. Specifically, the *tibialis anterior* corticospinal excitability, the silent period duration and the short-interval intracortical inhibition were not significant predictors of older adults’ temporal structure of stride-to-stride fluctuations.

Age-related brain changes have been suggested to impair motor control in older adults (Rosso et al., 2013). Importantly, several features of the human sensorimotor system deteriorate with ageing, which can alter the neural control of movement from a more automated (i.e., spinal cord and subcortical regions) towards a more cognitive process (i.e., cortical regions; Fettrow et al., 2023). Accordingly, emerging evidence supports a link between cognitive function and motor control (Rosso et al., 2014). This latter group of authors showed that step length variability was associated with grey matter integrity, particularly in brain regions specific to memory (hippocampus) and executive function (anterior cingulate gyrus). These findings highlight that age-related changes in specific brain regions could potentially underlie the emergence of increased step length variability in older adults (Rosso et al., 2014). We further questioned if additional levels of the neural information pathway, specifically, the corticospinal tract, could possibly be related with gait’s temporal structure of variability.

Ageing has been associated with a change in corticospinal excitability in older adults, although findings are conflicting, weakening robust conclusions. Some studies have demonstrated older adults exhibit decreased CSE compared with young healthy adults (McGinley et al., 2010; Oliviero et al., 2006). However, other authors’ works revealed no age-related changes in CSE occurred (Rozand et al., 2019). It has also been suggested that age-related changes in the intracortical inhibitory mechanisms of the primary motor cortex are likely present, albeit contrasting findings have been purported (Swanson and Fling, 2018). Accordingly, Christie and Kamen (2014) observed SP duration to be shorter in older when compared with younger adults for the *tibialis anterior*, indicating lower inhibition in the older group. In agreement, Oliviero and colleagues (2006) also observed lower SP duration in older adults for the *first dorsal interosseus* muscle, although observing no age-related changes for SICI. Contrarily, McGinley and colleagues (2010) demonstrated older adults exhibited an increase of SP duration and SICI for the *flexor carpi radialis*, denoting increased cortical inhibition. It appears that the involvement of the corticospinal tract in movement regulation remains a topic of considerable debate, underscoring the need for further research, particularly of studies exploring diverse motor tasks, different muscles of interest, and employing longitudinal designs, aimed at strengthening the robustness and generalizability of current conclusions.

A possible explanation of our findings could be related to the automatic regulation of human walking. Locomotion is fundamentally driven by spinal interneuronal circuits known as central pattern generators (CPGs), which produce the core motor rhythm underlying gait. These spinal networks adapt the locomotor output in response to proprioceptive and cutaneous afferent input, working together with descending information from the brainstem and cerebral cortex (Takakusaki, 2013). Interactions among the basal ganglia, cerebellum, and brainstem contribute to the automatic regulation of muscle tone and rhythmic limb activity without the need for conscious control (Middleton and Strick, 2000). Such automated regulation of gait may reduce the required involvement of the corticospinal tract in the modulation of stride-to-stride fluctuations, potentially helping to explain our findings. Given that automatic locomotor regulation relies heavily on brainstem and spinal circuitry, it is plausible that descending pathways originating from the brainstem, particularly the reticulospinal tract, may contribute more substantially to the control of steady-state gait than traditionally recognized (Takakusaki, 2013; Takakusaki et al., 2016). However, these mechanisms were not directly assessed in the present study and should therefore be considered hypotheses for future investigation rather than established explanations for the observed findings.

Accordingly, the reticulospinal tract is considered a major descending pathway involved in locomotion and postural control, particularly through the regulation of muscle tone during gait (Takakusaki et al., 2016). Unlike the corticospinal tract, the reticulospinal tract projects bilaterally within the spinal cord, innervating larger muscle groups and contributing predominantly to gross motor function (Akalu et al., 2023). Therefore, its role during gait may be more substantial than conventionally assumed. This notion is supported by studies in individuals with spinal cord injury showing increased reticulospinal excitability as a compensatory mechanism to preserve or restore motor function in the presence of reduced corticospinal drive (Baker and Perez, 2017; Sangari and Perez, 2019). While it appears the reticulospinal tract plays an important role in the recovery process following injury or disease, its contribution to gait’s motor control could also be heightened in an ageing population, further suggesting a shift toward greater reliance on reticulospinal pathways during locomotion. This interpretation is consistent with the impaired gait adaptability observed in our cohort (αISIs = 0.79 ± 0.15), which falls below the optimal threshold proposed to distinguish healthy young and older adults (Ravi et al., 2020). In contrast, age-related changes in corticospinal drive become particularly evident during gait tasks requiring greater conscious control, such as split-belt treadmill walking (Sato and Choi, 2022) and visually guided walking (Sato and Choi, 2023). These findings may suggest that corticospinal involvement is task-dependent, potentially becoming more relevant when locomotion requires increased voluntary adaptation and sensorimotor integration. However, the present study cannot determine whether corticospinal involvement differs between static and gait-related motor states. As an alternative explanation, it could also be that the age-related changes observed in stride-to-stride fluctuations are affecting, specifically, modifications in the cortico-cortical connections between premotor regions and the motor cortex. For example, the pathway between the cortico-subcortical connections between the premotor regions (inferior frontal gyrus) and the subthalamic nucleus has been linked to a higher rate of falls (Simon et al., 2024). Thus, our findings raise the possibility that the regulation of gait adaptability in highly automated walking tasks may rely less on corticospinal mechanisms, warranting a broader investigation of other neural structures and connections.

Human walking is organised and adjusted through spinal and supraspinal sensorimotor networks (Takakusaki, 2017). However, during walking, inherent and external challenges arise, which are particularly exacerbated in older individuals (Lipsitz and Goldberger, 1992; Manor and Lipsitz, 2013). To achieve steady gait, especially when facing the above-mentioned challenges, natural stride-to-stride fluctuations emerge, highlighting a key characteristic of human physiological signals: longe-range correlations, i.e., one stride-interval is correlated with stride intervals at distant time points (Ravi et al., 2020). This specific feature of human physiology is central to our rationale as longe-range correlations of stride intervals have been proposed to reflect functional interactions among central and peripheral schemes involved in the coordination of the musculoskeletal system, operating at different spatiotemporal scales within the body (Goldberger et al., 2002; Hausdorff, 2007; Ravi et al., 2020). Moreover, it has even been suggested that the level of persistence of these long-range correlations could reflect the level of rigidity of gait control by the upper centres (Dingwell et al., 2010), with anti-persistent long-range correlations possibly indicating a tight regulation of movement, rendering the system less adaptable (Ravi et al., 2020). Here, we demonstrate our sample of older adults exhibited a breakdown of their stride-to-stride fluctuations’ temporal organization towards less persistent long-range correlations, in agreement with Ravi and colleagues' (2020) optimal threshold for differentiating young and older adults (αISIs = 0.86). Nonetheless, this breakdown was not significantly predicted by the excitatory and inhibitory properties of the corticospinal tract.

While gait stride-to-stride fluctuations’ neurophysiological underlying mechanisms are currently unknown, they are speculated to arise from the complex interaction between our neuromuscular system and subsystems (Van Orden et al., 2005). Another potential explanation for our outcomes pertains to the hypothesis that the information from the corticospinal tract alone does not equate to predicting the level of adaptability of one’s gait, whereas if we consider this information together with other peripheral and central mechanisms, we could theoretically be able to observe different outcomes. In fact, several central and peripheral factors can influence the walking adaptation process (Spedden et al., 2019) and the breakdown of stride-to-stride fluctuations’ temporal structure can result from a progressive deterioration in the functioning elements of a given system or decrease in the number of interactions between these systems (Goldberger et al., 2002) which weaken an individual’s adaptive abilities (Lipsitz and Goldberger, 1992; Stergiou et al., 2006). For example, the integration of sensory information, which plays a crucial role in motor control, particularly during walking, as it helps drive the active motoneurons, contributes to corrections following sudden perturbations, and provides essential information that help the brain to update and improve future movements (Nielsen, 2003), has been shown to be compromised in older adults (Fettrow et al., 2023; Seidler et al., 2010). Age-related volume differences have also been observed in subcortical structures, such as the thalamus, caudate, putamen, pallidum, hippocampus, amygdala, among others (Goodro et al., 2012; Walhovd et al., 2011), which may be related with impaired motor control. When considering lower levels of the neural information pathway, ageing has also been linked with a neurodegenerative process of the spinal cord, primarily related to the loss of spinal motor neurons due to apoptosis of the neural cells (Borzuola et al., 2020). Moreover, ageing is also accompanied by a progressive loss of motor units, which might be associated with several neuromuscular adaptations in older adults, such as the decrease in motor units discharge rates and slower contractile properties (Dalton et al., 2010; Kidgell and Pearce, 2020; McNeil and Rice, 2018). Thus, a deeper understanding of the mechanisms underlying gait adaptability is essential and future research should investigate the contribution of additional levels of the neural information pathway towards the muscles to produce movement on the temporal structure of stride-to-stride fluctuations.

The current findings provide valuable knowledge regarding the understanding of the neural correlates of gait’s motor control process. Nonetheless, it is important to consider that walking on an instrumented treadmill may perturb the participant’s ability to walk freely, as in real life scenarios, even with proper familiarisation. Thus, in the future, the incorporation of more ecological contexts, such as walking outdoors, could potentially provide a more organic alternative. Additionally, an important methodological consideration pertains to the fact that the neuromuscular assessment was performed during a submaximal isometric ankle dorsiflexion task, and not during the actual gait task. This neuromuscular assessment methodology has been widely described in the literature (Pearce et al., 2023; Siddique et al., 2025a; Siddique et al., 2025b). Nevertheless, corticospinal excitability and intracortical inhibitory processes are strongly task dependent, and measures obtained during a static contraction may not fully represent their contribution during dynamic locomotion. Even though walking is characterised as a horizontal translation of the body in space, it also causes vertical displacement which may hinder the reliability of the TMS application (Mate, 2022). Moreover, the TMS application during gait could also draw attention on the walking task which typically requires little, or no attention (Mate, 2022), and the stepping gait pattern could be synchronised to external rhythmic clicking of TMS resulting in walking that is not free (Roerdink et al., 2011). Therefore, the absence of significant associations between the corticospinal measures assessed in the present study and the temporal structure of stride-to-stride fluctuations should be interpreted cautiously, as these measures may not adequately capture corticospinal activity underlying gait-specific motor control. Future studies combining gait-related measures of corticospinal excitability with assessments of stride-to-stride temporal structure may provide a more direct test of the contribution of corticospinal mechanisms to locomotor adaptability. Finally, the relatively small sample size and the absence of covariate adjustment should be considered when interpreting the present findings, as these factors may limit statistical power and the ability to account for potential between-participant differences that could influence the observed associations.

Finally, the present study’s findings provide evidence that corticospinal excitatory and inhibitory properties of the *tibialis anterior* do not significantly predict the temporal organization of stride-to-stride fluctuations during steady-state walking in older adults. This supports the notion that gait adaptability likely emerges from distributed and multilevel interactions among spinal, subcortical, and peripheral mechanisms, rather than from corticospinal contributions alone. Future research should therefore adopt more integrative and task-specific approaches to better elucidate the neural mechanisms underlying age-related changes in locomotor adaptability.

## Conclusion

The present study investigated whether the temporal structure of stride-to-stride fluctuations in older adults is explained by corticospinal drive to the lower limb, as indexed by corticospinal excitability, silent period duration, and short-interval intracortical inhibition of the *tibialis anterior*. Contrary to our hypothesis, none of these neurophysiological measures significantly predicted the fractal scaling exponent of inter-stride intervals, and the overall regression model was not statistically significant. These findings suggest that the temporal organization of gait variability in older adults is not significantly associated with corticospinal or intracortical excitatory and inhibitory properties. Rather, the results support the premise that stride-to-stride dynamics emerge from distributed and multilevel control processes that extend beyond corticospinal contributions alone. The regulation of gait variability likely reflects the integrated function of spinal, cortical and subcortical networks, as well as peripheral sensory feedback, whose interactions collectively shape locomotor adaptability. In this context, corticospinal measures obtained in isolated laboratory conditions may not capture the task-dependent and network-level dynamics that govern real-world gait motor control, warranting future and more comprehensive approaches to grasp the neural determinants of gait complexity in ageing.

## Data Availability

The raw data supporting the conclusions of this article will be made available by the authors, upon reasonable request. Requests to access the datasets should be directed to jvaz@egasmoniz.edu.pt.

## References

[B1] Akalu Y. Frazer A. K. Howatson G. Pearce A. J. Siddique U. Rostami M. (2023). Identifying the role of the reticulospinal tract for strength and motor recovery: a scoping review of nonhuman and human studies. Physiol. Rep. 11 (14), e15765. 10.14814/phy2.15765 37474275 PMC10359156

[B2] Baker S. N. Perez M. A. (2017). Reticulospinal contributions to gross hand function after human Spinal cord injury. J. Neurosci. 37 (40), 9778–9784. 10.1523/JNEUROSCI.3368-16.2017 28871033 PMC5628413

[B3] Barthelemy D. Nielsen J. B. (2010). Corticospinal contribution to arm muscle activity during human walking: cortical involvement in rhythmic arm movement. J. Physiology 588 (6), 967–979. 10.1113/jphysiol.2009.185520 PMC2849962 20123782

[B4] Barthélemy D. Grey M. J. Nielsen J. B. Bouyer L. (2011). Involvement of the corticospinal tract in the control of human gait. Prog. Brain Res. 192, 181–197. 10.1016/B978-0-444-53355-5.00012-9 21763526

[B5] Barthélemy D. Alain S. Grey M. J. Nielsen J. B. Bouyer L. J. (2012). Rapid changes in corticospinal excitability during force field adaptation of human walking. Exp. Brain Res. 217 (1), 99–115. 10.1007/s00221-011-2977-4 22246104

[B6] Bartsch R. P. Liu K. K. L. Bashan A. Ivanov P.Ch. (2015). Network physiology: how organ systems dynamically interact. PLOS ONE 10 (11), e0142143. 10.1371/journal.pone.0142143 26555073 PMC4640580

[B7] Borzuola R. Giombini A. Torre G. Campi S. Albo E. Bravi M. (2020). Central and peripheral neuromuscular adaptations to ageing. J. Clin. Med. 9 (3), 741. 10.3390/jcm9030741 32182904 PMC7141192

[B8] Buzzi U. H. Stergiou N. Kurz M. J. Hageman P. A. Heidel J. (2003). Nonlinear dynamics indicates aging affects variability during gait. Clin. Biomech. 18 (5), 435–443. 10.1016/S0268-0033(03)00029-9 12763440

[B9] Cavanaugh J. T. Kelty-Stephen D. G. Stergiou N. (2017). Multifractality, interactivity, and the adaptive capacity of the human movement System: a perspective for advancing the conceptual basis of neurologic physical therapy. J. Neurologic Phys. Ther. 41 (4), 245–251. 10.1097/NPT.0000000000000199 28834791 PMC5676558

[B10] Christie A. Kamen G. (2014). Cortical inhibition is reduced following short-term training in young and older adults. AGE 36 (2), 749–758. 10.1007/s11357-013-9577-0 23943112 PMC4039252

[B11] Colón-Emeric C. S. McDermott C. L. Lee D. S. Berry S. D. (2024). Risk assessment and prevention of Falls in older community-dwelling adults: a review. JAMA 331 (16), 1397. 10.1001/jama.2024.1416 38536167 PMC12224174

[B12] Dalton B. H. Jakobi J. M. Allman B. L. Rice C. L. (2010). Differential age‐related changes in motor unit properties between elbow flexors and extensors. Acta Physiol. 200 (1), 45–55. 10.1111/j.1748-1716.2010.02100.x 20158505

[B13] Damouras S. Chang M. D. Sejdić E. Chau T. (2010). An empirical examination of detrended fluctuation analysis for gait data. Gait and Posture 31 (3), 336–340. 10.1016/j.gaitpost.2009.12.002 20060298

[B14] Damron L. A. Dearth D. J. Hoffman R. L. Clark B. C. (2008). Quantification of the corticospinal silent period evoked *via* transcranial magnetic stimulation. J. Neurosci. Methods 173 (1), 121–128. 10.1016/j.jneumeth.2008.06.001 18588914

[B15] Devanne H. Lavoie B. A. Capaday C. (1997). Input-output properties and gain changes in the human corticospinal pathway. Exp. Brain Res. 114 (2), 329–338. 10.1007/PL00005641 9166922

[B16] Dingwell J. B. John J. Cusumano J. P. (2010). Do humans optimally exploit redundancy to control step variability in walking? PLoS Comput. Biol. 6 (7), e1000856. 10.1371/journal.pcbi.1000856 20657664 PMC2904769

[B17] Fettrow T. Hupfeld K. Hass C. Pasternak O. Seidler R. (2023). Neural correlates of gait adaptation in younger and older adults. Sci. Rep. 13 (1), 3842. 10.1038/s41598-023-30766-x 36890163 PMC9995534

[B18] Goldberger A. L. Amaral L. A. N. Hausdorff J. M. Ivanov P.Ch. Peng C.-K. Stanley H. E. (2002). Fractal dynamics in physiology: alterations with disease and aging. Proc. Natl. Acad. Sci. 99 (Suppl. l_1), 2466–2472. 10.1073/pnas.012579499 11875196 PMC128562

[B19] Goodro M. Sameti M. Patenaude B. Fein G. (2012). Age effect on subcortical structures in healthy adults. Psychiatry Res. Neuroimaging 203 (1), 38–45. 10.1016/j.pscychresns.2011.09.014 22863654 PMC3444666

[B20] Harbourne R. T. Stergiou N. (2009). Movement variability and the use of nonlinear tools: principles to guide physical therapist practice. Phys. Ther. 89 (3), 267–282. 10.2522/ptj.20080130 19168711 PMC2652347

[B21] Hausdorff J. M. (2005). Gait variability: methods, modeling and meaning. J. NeuroEngineering Rehabilitation 2 (1), 19. 10.1186/1743-0003-2-19 16033650 PMC1185560

[B22] Hausdorff J. M. (2007). Gait dynamics, fractals and falls: finding meaning in the stride-to-stride fluctuations of human walking. Hum. Mov. Sci. 26 (4), 555–589. 10.1016/j.humov.2007.05.003 17618701 PMC2267927

[B23] Hausdorff J. M. Mitchell S. L. Firtion R. Peng C. K. Cudkowicz M. E. Wei J. Y. (1997). Altered fractal dynamics of gait: reduced stride-interval correlations with aging and Huntington’s disease. J. Appl. Physiology 82 (1), 262–269. 10.1152/jappl.1997.82.1.262 9029225

[B24] Hausdorff J. M. Rios D. A. Edelberg H. K. (2001). Gait variability and fall risk in community-living older adults: a 1-year prospective study. Archives Phys. Med. Rehabilitation 82 (8), 1050–1056. 10.1053/apmr.2001.24893 11494184

[B25] Hausdorff J. M. Schaafsma J. D. Balash Y. Bartels A. L. Gurevich T. Giladi N. (2003). Impaired regulation of stride variability in Parkinson’s disease subjects with freezing of gait. Exp. Brain Res. 149 (2), 187–194. 10.1007/s00221-002-1354-8 12610686

[B26] Hermens H. J. Freriks B. Disselhorst-Klug C. Rau G. (2000). Development of recommendations for SEMG sensors and sensor placement procedures. J. Electromyogr. Kinesiol. 10 (5), 361–374. 10.1016/S1050-6411(00)00027-4 11018445

[B27] Hirano M. Kubota S. Tanabe S. Koizume Y. Funase K. (2015). Interactions among learning stage, retention, and primary motor cortex excitability in motor skill learning. Brain Stimul. 8 (6), 1195–1204. 10.1016/j.brs.2015.07.025 26256670

[B28] Hirano M. Kubota S. Koizume Y. Funase K. (2018). Acquisition of motor memory determines the interindividual variability of learning-induced plasticity in the primary motor cortex. J. Appl. Physiology 125 (4), 990–998. 10.1152/japplphysiol.00470.2018 29975602

[B29] Hunt N. McGrath D. Stergiou N. (2014). The influence of auditory-motor coupling on fractal dynamics in human gait. Sci. Rep. 4 (1), 5879. 10.1038/srep05879 25080936 PMC4118321

[B30] Keel J. C. Smith M. J. Wassermann E. M. (2001). A safety screening questionnaire for transcranial magnetic stimulation. Clin. Neurophysiol. 112 (4), 720. 10.1016/S1388-2457(00)00518-6 11332408

[B31] Kidgell D. J. Pearce A. J. (2010). Corticospinal properties following short-term strength training of an intrinsic hand muscle. Hum. Mov. Sci. 29 (5), 631–641. 10.1016/j.humov.2010.01.004 20400192

[B32] Kidgell D. J. Pearce A. (2020). Principles of Exercise Neuroscience. Newcastle upon Tyne: Cambridge Scholars Publishing.

[B33] Landi F. Liperoti R. Russo A. Giovannini S. Tosato M. Capoluongo E. (2012). Sarcopenia as a risk factor for falls in elderly individuals: results from the ilSIRENTE study. Clin. Nutr. 31 (5), 652–658. 10.1016/j.clnu.2012.02.007 22414775

[B34] Lipsitz L. Goldberger A. (1992). Loss of ‘Complexity’ and aging. JAMA 267 (13), 1806–1809. 10.1001/jama.1992.03480130122036 1482430

[B35] Lipsitz L. A. Manor B. Habtemariam D. Iloputaife I. Zhou J. Travison T. G. (2018). The pace and prognosis of peripheral sensory loss in advanced age: association with gait speed and falls. BMC Geriatr. 18 (1), 274. 10.1186/s12877-018-0970-5 30419857 PMC6233369

[B36] Manor B. Lipsitz L. A. (2013). Physiologic complexity and aging: implications for physical function and rehabilitation. Prog. Neuro-Psychopharmacology Biol. Psychiatry 45, 287–293. 10.1016/j.pnpbp.2012.08.020 22985940 PMC3568237

[B37] Manor B. Costa M. D. Hu K. Newton E. Starobinets O. Kang H. G. (2010). Physiological complexity and system adaptability: evidence from postural control dynamics of older adults. J. Appl. Physiology 109 (6), 1786–1791. 10.1152/japplphysiol.00390.2010 20947715 PMC3006415

[B38] Mason J. Frazer A. K. Avela J. Pearce A. J. Howatson G. Kidgell D. J. (2020). Tracking the corticospinal responses to strength training. Eur. J. Appl. Physiology 120 (4), 783–798. 10.1007/s00421-020-04316-6 32060740

[B39] Mate K. K. V. (2022). Transcranial magnetic stimulation during gait: a review of methodological and technological challenges. Neurol. India 70 (4), 1448–1453. 10.4103/0028-3886.355114 36076642

[B40] McGinley M. Hoffman R. L. Russ D. W. Thomas J. S. Clark B. C. (2010). Older adults exhibit more intracortical inhibition and less intracortical facilitation than young adults. Exp. Gerontol. 45 (9), 671–678. 10.1016/j.exger.2010.04.005 20417265 PMC2926152

[B41] McNeil C. J. Rice C. L. (2018). Neuromuscular adaptations to healthy aging. Appl. Physiology, Nutr. Metabolism 43 (11), 1158–1165. 10.1139/apnm-2018-0327 29986151

[B42] Middleton F. Strick P. (2000). Basal ganglia and cerebellar loops: motor and cognitive circuits. Brain Res. Rev. 31 (2–3), 236–250. 10.1016/S0165-0173(99)00040-5 10719151

[B43] Mrachacz-Kersting N. Stevenson A. J. T. Ziemann U. (2021). Short-interval intracortical inhibition and facilitation targeting upper and lower limb muscles. Sci. Rep. 11 (1), 21993. 10.1038/s41598-021-01348-6 34754010 PMC8578654

[B44] Nielsen J. B. (2003). How we walk: central control of muscle activity during human walking. Neurosci. 9 (3), 195–204. 10.1177/1073858403009003012 15065815

[B45] Oliviero A. Profice P. Tonali P. A. Pilato F. Saturno E. Dileone M. (2006). Effects of aging on motor cortex excitability. Neurosci. Res. 55 (1), 74–77. 10.1016/j.neures.2006.02.002 16584795

[B46] Paci M. Di Cosmo G. Perrucci M. G. Ferri F. Costantini M. (2021). Cortical silent period reflects individual differences in action stopping performance. Sci. Rep. 11 (1), 15158. 10.1038/s41598-021-94494-w 34312403 PMC8313697

[B47] Pearce A. J. Kidgell D. J. Frazer A. K. Rist B. Tallent J. (2023). Evidence of altered corticomotor inhibition in older adults with a history of repetitive neurotrauma. A transcranial magnetic stimulation study. J. Neurological Sci. 453, 120777. 10.1016/j.jns.2023.120777 37677860

[B48] Peng C.-K. Havlin S. Stanley H. E. Goldberger A. L. (1995). Quantification of scaling exponents and crossover phenomena in nonstationary heartbeat time series. Chaos An Interdiscip. J. Nonlinear Sci. 5 (1), 82–87. 10.1063/1.166141 11538314

[B49] Perera C. K. Gopalai A. A. Ahmad S. A. Gouwanda D. (2021). Muscles affecting minimum toe clearance. Front. Public Health 9, 612064. 10.3389/fpubh.2021.612064 34136448 PMC8200481

[B50] Petersen N. T. Butler J. E. Marchand‐Pauvert V. Fisher R. Ledebt A. Pyndt H. S. (2001). Suppression of EMG activity by transcranial magnetic stimulation in human subjects during walking. J. Physiology 537 (2), 651–656. 10.1111/j.1469-7793.2001.00651.x 11731595 PMC2278954

[B51] Petersen T. H. Willerslev‐Olsen M. Conway B. A. Nielsen J. B. (2012). The motor cortex drives the muscles during walking in human subjects. J. Physiology 590 (10), 2443–2452. 10.1113/jphysiol.2012.227397 22393252 PMC3424763

[B52] Ravi D. K. Marmelat V. Taylor W. R. Newell K. M. Stergiou N. Singh N. B. (2020). Assessing the temporal Organization of Walking Variability: a Systematic Review and Consensus Guidelines on detrended fluctuation analysis. Front. Physiology 11, 562. 10.3389/fphys.2020.00562 32655400 PMC7324754

[B53] Ravi D. K. Bartholet M. Skiadopoulos A. Kent J. A. Wickstrom J. Taylor W. R. (2021). Rhythmic auditory stimuli modulate movement recovery in response to perturbation during locomotion. J. Exp. Biol. 224 (5), jeb237073. 10.1242/jeb.237073 33536309 PMC7938806

[B54] Roeder L. Boonstra T. W. Smith S. S. Kerr G. K. (2018). Dynamics of corticospinal motor control during overground and treadmill walking in humans. J. Neurophysiology 120 (3), 1017–1031. 10.1152/jn.00613.2017 29847229

[B55] Roerdink M. Bank P. J. M. Peper C. Beek P. J. (2011). Walking to the beat of different drums: practical implications for the use of acoustic rhythms in gait rehabilitation. Gait and Posture, 33(4), 690–694. 10.1016/j.gaitpost.2011.03.001 21454077

[B56] Rossini P. M. Burke D. Chen R. Cohen L. G. Daskalakis Z. Di Iorio R. (2015). Non-invasive electrical and magnetic stimulation of the brain, spinal cord, roots and peripheral nerves: basic principles and procedures for routine clinical and research application. An updated report from an I.F.C.N. Committee. Clin. Neurophysiol. 126 (6), 1071–1107. 10.1016/j.clinph.2015.02.001 25797650 PMC6350257

[B57] Rosso A. L. Studenski S. A. Chen W. G. Aizenstein H. J. Alexander N. B. Bennett D. A. (2013). Aging, the central nervous System, and mobility. Journals Gerontology Ser. A 68 (11), 1379–1386. 10.1093/gerona/glt089 23843270 PMC3805295

[B58] Rosso A. L. Olson Hunt M. J. Yang M. Brach J. S. Harris T. B. Newman A. B. (2014). Higher step length variability indicates lower gray matter integrity of selected regions in older adults. Gait and Posture 40 (1), 225–230. 10.1016/j.gaitpost.2014.03.192 24792638 PMC4071448

[B59] Rozand V. Senefeld J. W. Sundberg C. W. Smith A. E. Hunter S. K. (2019). Differential effects of aging and physical activity on corticospinal excitability of upper and lower limb muscles. J. Neurophysiology 122 (1), 241–250. 10.1152/jn.00077.2019 31091158 PMC6689774

[B60] Sangari S. Perez M. A. (2019). Imbalanced corticospinal and reticulospinal contributions to spasticity in humans with spinal cord injury. J. Neurosci. 39 (40), 7872–7881. 10.1523/JNEUROSCI.1106-19.2019 31413076 PMC6774405

[B61] Sato S. D. Choi J. T. (2022). Corticospinal drive is associated with temporal walking adaptation in both healthy young and older adults. Front. Aging Neurosci. 14, 920475. 10.3389/fnagi.2022.920475 36062156 PMC9436318

[B62] Sato S. D. Choi J. T. (2023). Reduced corticospinal drive and inflexible temporal adaptation during visually guided walking in older adults. J. Neurophysiology 130 (6), 1508–1520. 10.1152/jn.00078.2023 37937342 PMC10994519

[B63] Seidler R. D. Bernard J. A. Burutolu T. B. Fling B. W. Gordon M. T. Gwin J. T. (2010). Motor control and aging: links to age-related brain structural, functional, and biochemical effects. Neurosci. and Biobehav. Rev. 34 (5), 721–733. 10.1016/j.neubiorev.2009.10.005 19850077 PMC2838968

[B64] Siddique U. Frazer A. K. Tallent J. Hayman O. Andrushko J. Ahtiainen J. P. (2025a). Acute corticospinal and reticulospinal responses to strength training in ageing. Neurobiol. Aging 153, 49–62. 10.1016/j.neurobiolaging.2025.06.007 40578297

[B65] Siddique U. Frazer A. K. Tallent J. Hayman O. Andrushko J. Stock M. S. (2025b). Novel evidence of age-related cortical and subcortical constraints in cross-education. GeroScience 48, 5485–5508. 10.1007/s11357-025-01886-1 41138058 PMC13574755

[B66] Simon C. Bolton D. A. E. Meaney J. F. Kenny R. A. Simon V. A. De Looze C. (2024). White matter fibre density in the brain’s inhibitory control network is associated with falling in low activity older adults. Eur. J. Neurosci. 59 (12), 3184–3202. 10.1111/ejn.16327 38638001

[B67] Spedden M. E. Nielsen J. B. Geertsen S. S. (2018). Oscillatory corticospinal activity during static contraction of ankle muscles is reduced in healthy old *versus* young adults. Neural Plast. 2018, 1–13. 10.1155/2018/3432649 29853842 PMC5944232

[B68] Spedden M. E. Choi J. T. Nielsen J. B. Geertsen S. S. (2019). Corticospinal control of normal and visually guided gait in healthy older and younger adults. Neurobiol. Aging 78, 29–41. 10.1016/j.neurobiolaging.2019.02.005 30852367

[B69] Stergiou N. (2018). “Fractals,” in Nonlinear Analysis for Human Movement Variability (Boca Raton: CRC Press), 261–300. 10.1201/9781315370651

[B70] Stergiou N. Decker L. M. (2011). Human movement variability, nonlinear dynamics, and pathology: is there a connection? Hum. Mov. Sci. 30 (5), 869–888. 10.1016/j.humov.2011.06.002 21802756 PMC3183280

[B71] Stergiou N. Harbourne R. T. Cavanaugh J. T. (2006). Optimal movement variability: a new theoretical perspective for neurologic physical therapy. J. Neurologic Phys. Ther. 30 (3), 120–129. 10.1097/01.NPT.0000281949.48193.d9 17029655

[B72] Swanson C. W. Fling B. W. (2018). Associations between gait coordination, variability and motor cortex inhibition in young and older adults. Exp. Gerontol. 113, 163–172. 10.1016/j.exger.2018.10.002 30296454

[B73] Takakusaki K. (2013). Neurophysiology of gait: from the spinal cord to the frontal lobe. Mov. Disord. 28 (11), 1483–1491. 10.1002/mds.25669 24132836

[B74] Takakusaki K. (2017). Functional neuroanatomy for posture and gait control. J. Mov. Disord. 10 (1), 1–17. 10.14802/jmd.16062 28122432 PMC5288669

[B75] Takakusaki K. Chiba R. Nozu T. Okumura T. (2016). Brainstem control of locomotion and muscle tone with special reference to the role of the mesopontine tegmentum and medullary reticulospinal systems. J. Neural Transm. 123 (7), 695–729. 10.1007/s00702-015-1475-4 26497023 PMC4919383

[B76] Tallent J. Goodall S. Hortobágyi T. St Clair Gibson A. French D. N. Howatson G. (2012). Repeatability of corticospinal and spinal measures during lengthening and shortening contractions in the Human Tibialis anterior muscle. PLoS ONE 7 (4), e35930. 10.1371/journal.pone.0035930 22563418 PMC3338551

[B77] Thompson A. K. Lapallo B. Duffield M. Abel B. M. Pomerantz F. (2011). Repetitive common peroneal nerve stimulation increases ankle dorsiflexor motor evoked potentials in incomplete spinal cord lesions. Exp. Brain Res. 210 (1), 143–152. 10.1007/s00221-011-2607-1 21360230

[B78] Van Orden G. C. Holden J. G. Turvey M. T. (2005). Human cognition and 1/f scaling. J. Exp. Psychol. General 134 (1), 117–123. 10.1037/0096-3445.134.1.117 15702967

[B81] Vaz J. R. Cortes N. Gomes J. S. Jordão S. Stergiou N. (2024). Stride-to-stride fluctuations and temporal patterns of muscle activity exhibit similar responses during walking to variable visual cues. Journal of biomechanics 164, 111972. 10.1016/j.jbiomech.2024.111972 38330885 PMC11034849

[B79] Walhovd K. B. Westlye L. T. Amlien I. Espeseth T. Reinvang I. Raz N. (2011). Consistent neuroanatomical age-related volume differences across multiple samples. Neurobiol. Aging 32 (5), 916–932. 10.1016/j.neurobiolaging.2009.05.013 19570593 PMC4040218

[B80] Watson N. L. Sutton-Tyrrell K. Youk A. O. Boudreau R. M. Mackey R. H. Simonsick E. M. (2011). Arterial stiffness and gait speed in older adults with and without peripheral arterial disease. Am. J. Hypertens. 24 (1), 90–95. 10.1038/ajh.2010.193 20940711 PMC3290653

