# Supplementary material for: Corticospinal function does not predict stride-to-stride gait dynamics in older adults
Source: Front Netw Physiol. 2026 Oct 9;6:1946459. doi: 10.3389/fnetp.2026.1946459 (PMC13714503; doi:10.3389/fnetp.2026.1946459)
Supplement: Supplementary file 1 [file Table1.docx]

|  | **Mean ± SD** | **95 % CI** |
| --- | --- | --- |
| **Gait Assessment** |  |  |
| αISIs (au) | 0.79 ± 0.15 | [0.74, 0.85] |
| r^2^ (au) | 0.94 ± .03 | [0.93, 0.95] |
| ISIs length (nº of strides) | 628 ± 63.1 | [605, 651] |
| PWS (ms^-1^) | 1.16 ± 0.17 | [1.10, 1.22] |
| **Neuromuscular Assessment** |  |  |
| MVC (Nm^-1^) | 35.7 ± 9.30 | [32.3, 39.1] |
| AMT (% MSO) | 40.4 ± 6.46 | [38.0, 42.8] |
| CSE (mV) | 0.91 ± 0.38 | [0.78, 1.05] |
| SP (ms) | 127 ± 19.5 | [120, 134] |
| SICI (%) | 91.8 ± 17.5 | [85.6, 97.9] |
